# ZBED6 binding motifs correlate with endogenous retroviruses and *Syncytin* genes

**DOI:** 10.1093/ve/veaa083

**Published:** 2020-11-06

**Authors:** Mats E Pettersson, Patric Jern

**Affiliations:** Science for Life Laboratory, Department of Medical Biochemistry and Microbiology, Uppsala University, Uppsala, Sweden

**Keywords:** ERV, ZBED6, motif, syncytin, transposon

## Abstract

Retroviruses have infiltrated vertebrate germlines for millions of years as inherited endogenous retroviruses (ERVs). Mammalian genomes host large numbers of ERVs and transposable elements (TEs), including retrotransposons and DNA transposons, that contribute to genomic innovation and evolution as coopted genes and regulators of diverse functions. To explore features distinguishing coopted ERVs and TEs from other integrations, we focus on the potential role of ZBED6 and repeated ERV domestication as repurposed *Syncytin* genes. The placental mammal-specific ZBED6 is a DNA transposon-derived transcription regulator and we demonstrate that its binding motifs are associated with distinct *Syncytin*s and that ZBED6 binding motifs are 2- to 3-fold more frequent in ERVs than in flanking DNA. Our observations suggest that ZBED6 could contribute an extended regulatory role of genomic expression, utilizing ERVs as platforms for genomic innovation and evolution.

## 1. Introduction

Mammalian genomes contain large numbers of repetitive sequences deriving from transposable elements (TEs), including DNA and retrotransposons. Additionally, retroviruses have infiltrated host germline cells during millions of years and have become inherited endogenous retroviruses (ERVs) (Jern and Coffin 2008). These TEs and ERVs provide substrate for genomic innovation and evolution as coopted genes that have been repurposed by the host as regulators of diverse functions (Hayward et al. 2013; Chuong et al. 2017) in a process referred to as molecular domestication (Volff 2006; Feschotte and Pritham 2007; Sinzelle et al. 2009). The extent to which ERVs are repurposed by the host and which discriminating features are facilitating this genomic novelty compared to the vast majority of non-coopted ERVs is pertinent questions for improved understanding of mammalian evolution and host pathogen associations.

Syncytin proteins, which are involved in trophoblast cell–cell fusion and formation of syncytia during placental development, are the results of multiple independent ERV domestications. In each case, the retroviral *env* gene has been repurposed from its normal function of producing surface and transmembrane proteins for retroviral and host cell membrane fusion (Lavialle et al. 2013). About ten non-orthologous ERVs in eight host lineages have been repurposed to contribute cell–cell fusion activities (Lavialle et al. 2013; Denner 2016). It is thus conceivable that the independent ERV domestications improved a primordial placental function and that these domestications were facilitated by mammalian-specific qualities that promoted cooption of certain ERVs with specific features distinguishing them from the vast pool of non-coopted ERVs in mammalian genomes.

*ZBED* (Zinc-finger BED-type domain containing) genes derive from domesticated DNA transposons and contribute diverse regulatory functions in vertebrate host genomes (Hayward et al. 2013). The mammalian-specific ZBED6 is shown to regulate host expression by binding to conserved motifs adjacent to host genes (Markljung et al. 2009) and is thus an intriguing candidate for further analysis as a potential cofactor to non-orthologous domesticated ERVs, *Syncytin* expression, and effects on placental development. ZBED6 is integrated into an intron of *ZC3H11A* (Zinc Finger CCCH-Type Containing 11A) (Markljung et al. 2009) and appears to be expressed from the same promoter as *ZC3H11A* already during embryo development, which is in line with temporal expression of ZBED6 as a potential regulator of *Syncytin* expression during, for instance, placenta formation.

Here, we explore the ERV catalogue (Sperber et al. 2007; Hayward et al. 2015) to assess sequence features in, or flanking, ten non-orthologous ERV-derived *Syncytins* in eight different host species, and compare with tens of thousands non-coopted ERVs to better understand common aspects of these gene domestications. Overall it is plausible that the coopted ERVs contain features influencing their expression that other ERVs lack, and we search for sequence motifs that could constitute such features with specific attention to those potentially associated with ZBED6 binding.

## 2. Results and discussion

Ten *Syncytin* loci in eight species genome assemblies were located and accessed from the UCSC genome browser (http://genome.ucsc.edu/), and screened for ZBED6 consensus binding motifs (5′-GCTCG-3′) (Markljung et al. 2009). Guided by the outcome of previous CHiPseq experiments (Markljung et al. 2009), we focused on 1,000 nt search distances up- and downstream start positions for *Syncytins* and ERVs (including detected 5′LTRs). We identified nine canonical ZBED6 binding motifs within 1,000 nt up- and downstream *Syncytin* starts in eight of ten genome assemblies (Table 1). The screening failed to identify ZBED6 binding motifs within 1,000 nt of the *Syncytin-Ten1* start, possibly due to the comparatively lower quality of the Tenrec assembly and annotation. Neither could we identify ZBED6 binding motifs associated with *Syncytin-Opo1*, which could be due to the mammalian-specific *ZBED6* gene missing from the genome, as it is not detectable in the Opossum assembly. ZBED6 binding motifs are therefore likely not under selective pressure in this host lineage. This is also in line with the favored mode of gestation in marsupials being an external pouch instead of the short-lived placenta (Cornelis et al. 2015; Guernsey et al. 2017). Overall, it is striking that 80 per cent of the non-orthologous domesticated ERV-derived *Syncytin* genes have ZBED6 binding motifs adjacent to their start positions.

**Table 1.** Syncytin loci and associated ZBED6 motifs.

Syncytin^a^	Reference	Locus	Genome	Species	Assembly^b^	Positions^c^	Strand	ZBED6-motifs^d^
Syncytin-1	Blond et al. (2000) and Mi et al. (2000)	ERVW1	Human	*Homo sapiens*	hg38	chr7:92468380-92477946	–	1
Syncytin-2	Blaise et al. (2003)	ERVFRD1	Human	*Homo sapiens*	hg38	chr6:11102489-11111725	–	1
Syncytin-A	Dupressoir et al. (2005)	SYNA	Mouse	*Mus musculus*	mm10	chr5:134558146-134560171	–	1
Syncytin-B	Dupressoir et al. (2005)	SYNB	Mouse	*Mus musculus*	mm10	chr14:69289344-69294299	–	1
Syncytin-Car1	Cornelis et al. (2012)	CAR1	Dog	*Canis familiaris*	canFam3	chr3:83844415-83845836	+	2
Syncytin-Mar1	Redelsperger et al. (2014)	MAR1	Squirrel	*Ictidomys tridecemlineatus*	speTri2	JH393329:6380706-6382478	–	1
Syncytin-Opo1	Cornelis et al. (2015)	OPO1	Opossum	*Monodelphis domestica*	monDom5	chr8:120625037-120643830	–	0
Syncytin-Ory1	Heidmann et al. (2009)	ORY1	Rabbit	*Oryctolagus cuniculus*	oryCun2	chr12:99949134-99950867	+	1
Syncytin-Rum1	Cornelis et al. (2013)	RUM1	Cattle	*Bos taurus*	BosTau8	chr13:78406494-78408957	–	1
Syncytin-Ten1	Cornelis et al. (2014)	TEN1	Tenrec	*Echniops telfairi*	echTel2	JH980362:6221096-6233249	–	0

a Coopted (non-orthologous) *Syncytin* loci assessed in this study.

b Assembly version accessed from the UCSC genome browser (http://genome.ucsc.edu/).

c Chromosomal positions analyzed here with focus on start and 1,000 nt flanking DNA.

d Identified ZBED6 binding motifs (5′-GCTCG-3′) within 1,000 nt up- and downstream *Syncytin* loci start positions.

Next, we screened the ERV catalogue (Hayward et al. 2015) for the eight species genome assemblies above, focusing on 1,000 nt flanking the start positions for ERVs (i.e. the 5′LTR, if detected) identified by the RetroTector software (Sperber et al. 2007), to establish the overall frequencies of ERVs and ZBED6 binding motifs (Table 2). From the eight genome assemblies, we identified 26,266 ERVs and also note low ERV counts in the Tenrec assembly, which could be explained by the reasoning above. From the identified ERVs, we found 10,016 ZBED6 binding motifs located within 1,000 nt of starting positions from 7,834 ERVs. It is thus approximately a 30 per cent chance to find ZBED6 binding motifs associated with ERVs in these genome assemblies, in contrast to the 80 per cent chance to find ZBED6 binding motifs inside equivalent intervals around domesticated ERV-derived *Syncytin* genes (Fisher *P* < 0.05). To explore motif-associated ERV relationships, we could search 6,498 of the 7,835 motif-associated ERVs against previous phylogenetic classifications (Hayward et al. 2015), observing that 153 motif-associated ERVs (of 542 ERVs in the phylogeny deriving from corresponding genome assemblies) located across the retroviral diversity at frequencies expected from overall ERV representation (about 37% *gamma-* and 31% *beta-like* ERVs, Fisher *P* > 0.4), and that *epsilon-like* ERVs classified mainly in the Opossum were fewer than expected (<10%). In addition, to narrow the scope and evaluating whether ZBED6 binding motifs were more likely associated with ERVs related to coopted *Syncytins* than associated with related non-coopted ERVs, we isolated *Syncytin-1* related ERVs from the human genome assembly (hg38). ZBED6 binding motifs were associated with 25 of 80 ERVs (31%) in this group, which is not significantly different from the overall expected 30 per cent frequency in Table 2 (Fisher *P* > 0.8). Together, these results demonstrate that motif-associated ERVs are found across the retroviral diversity and are not limited to specific subclades. However, this observation does not exclude potential contributions from other shared sequence features within motif-associated ERVs.

**Table 2.** ERVs and associated ZBED6 binding motifs.

Assembly	ERV.Tot^a^	ERV.Motifs^b^	Motifs^c^
hg38	4,101	1,050	1,331
mm10	9,338	3,583	4,663
canFam3	928	286	441
speTri2	1,980	728	977
monDom5	6,980	1,197	1,294
oryCun2	960	299	420
BosTau8	1,714	604	769
echTel2	265	87	121
∑	26,266	7,834	10,016

a Identified ERVs by RetroTector analysis (Sperber et al. 2007; Hayward et al. 2015).

b Number of ERVs associated with ZBED6 motifs (5′-GCTCG-3′) within 1,000 nt flanking ERV start positions.

c ZBED6 binding motif (5′-GCTCG-3′) counts within 1,000 nt flanking ERV start positions.

**Figure 1.**
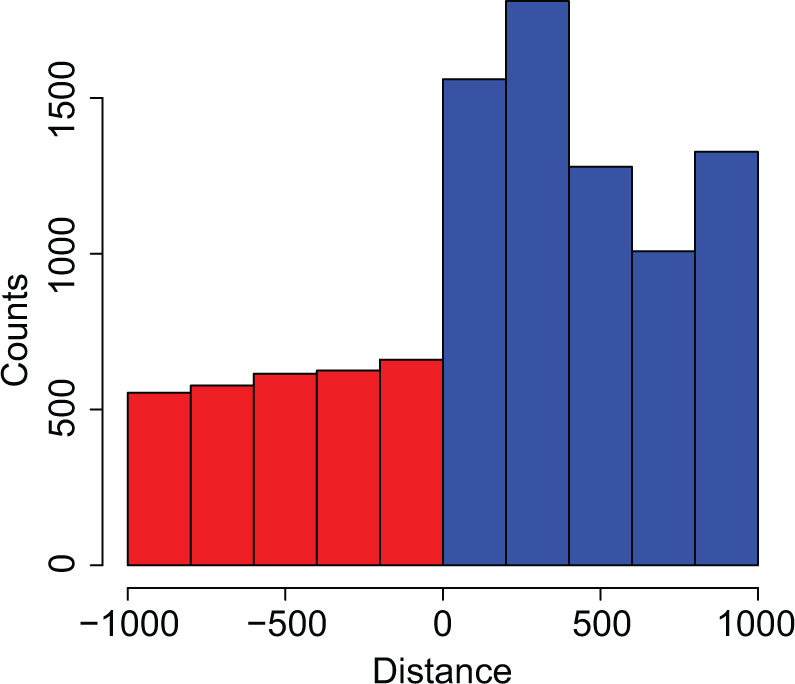
ERV associated ZBED6 binding motif (5′-GCTCG-3′) counts within 1,000 nt flanking ERV start positions. Negative distances (red) indicate canonical motif counts in upstream flanking DNA and positive distances (blue) indicate motif counts in 5′-ERV sequence.

Summarizing distances for ZBED6 binding motifs relative to ERV start positions identified by RetroTector (Sperber et al. 2007; Hayward et al. 2015), we note a striking increase in motifs immediately inside the ERV sequence, compared to upstream flanking DNA (Fig. 1). On average, the 5′-end of ERVs (including detected 5′LTRs) present about 2- to 3-fold more ZBED6 binding motifs compared to upstream flanking DNA, suggesting that these ERVs could serve as binding platforms for ZBED6-mediated regulation of transcription. These results present an intriguing connection with the higher-than-expected ZBED6 binding motif frequency associated with the repurposed ERV-derived *Syncytins* (80% compared to 30% for ERVs in general) that warrants further investigation.

To evaluate genomic effects of ZBED6 binding to ERVs, we accessed differential gene expression data from fetal muscle tissue samples generated by short-read RNA sequencing of ZBED6 wildtype and knockout mice (Younis et al. 2018). ZBED6 binding motif-associated ERVs, identified in the mouse genome assembly (mm10, Table 2), overlapped with two of fifty-seven significant mouse ZBED6 knockout differential expression genes (Ephb1, Dock3) (Younis et al. 2018), indicating that ERVs could provide docking platforms to ZBED6 for regulatory effects on adjacent gene expression. Next, we intersected all mapped reads with the 9,338 identified mouse ERV positions (assembly version mm10) of which 3,583 ERVs present 4,663 ZBED6 binding motifs (Table 2) and found that some loci were expressed while other ERVs did not yield any mappable reads, suggesting differential expression across the genome. Interestingly, among all expressed ZBED6 binding motif-associated ERVs across the genome, we found a significant 25 per cent increase in normalized ERV read counts in ZBED6 knockout mouse fetal muscle tissue samples compared to the wildtype equivalent (Wilcoxon paired rank test P: 1.1 × 10^−118^). This result agrees with previous indications that ZBED6 acts primarily as a regulatory repressor for adjacent gene expression (Markljung et al. 2009). Data suggest that ZBED6 could have additional negative regulatory function across the genome not associated with specific motifs in ERVs.

To summarize, we demonstrate that binding motifs for the domesticated DNA transposon derived and mammalian-specific ZBED6 are associated with ERVs across host genomes and are over-represented near coopted ERV-derived *Syncytin* genes. It is clear that ZBED6 binding motifs are not a strict requirement for regulating *Syncytin* expression during mammalian placenta formation, as ZBED6 knockout mice survive and produce litters (Younis et al. 2018). However, our bioinformatic screening suggests that ZBED6 binding motifs could contribute to effects on adjacent gene expression because of the significant 25 per cent difference in ERV expression between ZBED6 wildtype and knockout mice. Further confirmation of temporal effects of ZBED6-associated regulation of transcription during placenta formation requires additional samples, which are currently not available and thus out of scope for this study. It is possible that ZBED6 could have additional regulatory function across the genome not associated with specific motifs in ERVs, and additional studies along these lines with experimental confirmations are warranted.

In conclusion, we present observations justifying further investigations testing the hypothesis that domesticated TEs, such as the mammalian-specific ZBED6, perform regulatory functions in vertebrate host DNA and are able to utilize the abundance of ERVs and the binding motifs provided by those elements as substrate for this regulation, similarly to other recently demonstrated regulatory proteins such as TRIM28 (Fasching et al. 2015; Brattas et al. 2017), for tuning expression levels, genomic innovation and thus evolution. 
